# Flexible Graphene-Based S-Band Metasurface Conformal Array Antenna for UAV Platforms

**DOI:** 10.3390/ma19112404

**Published:** 2026-06-04

**Authors:** Jinling Li, Peng Li, Meng Zeng, Yitong Xin, Haoran Zu, Rongguo Song

**Affiliations:** 1 Hubei Engineering Research Center of RF-Microwave Technology and Application, Wuhan University of Technology, Wuhan 430070, China; lijl@whut.edu.cn (J.L.); lipeng871120@whut.edu.cn (P.L.); 347466@whu.edu.cn (M.Z.); xinyitong@whut.edu.cn (Y.X.); 2 School of Information Engineering, Wuhan University of Technology, Wuhan 430070, China

**Keywords:** graphene-assembled film, frequency selective surface, conformal antenna, S-band, UAV, wing-conformal array, beam scanning

## Abstract

There is a substantial demand for lightweight, low-profile, and conformal antenna integration on the wing platforms of unmanned aerial vehicles (UAVs). This paper presents an S-band (2–4 GHz) flexible conformal metasurface array antenna based on a highly conductive graphene-assembled film (GAF). The main contributions of this work are twofold. First, flexible and highly conductive GAF is used as the conductor together with a flexible polyimide (PI) dielectric substrate to form a GAF-based wing-conformal antenna configuration with a low-profile, lightweight, and easily conformal performance. Second, a GAF conformal antenna element is developed by combining a dipole antenna with a directive and reflective frequency selective surface (FSS), achieving effective control of the beam and stable directional radiation at 2.4 GHz. Full-wave simulations using CST Studio Suite show that the directive FSS narrows the feed beam, whereas the reflective FSS redirects and narrows the H-plane radiation. The simulated results show that the integrated wing-conformal antenna operates over 2.19–2.65 GHz and achieves a gain of 4.65 dBi at 2.4 GHz. The measurement results indicate that the GAF conformal antenna and 1 × 4 GAF conformal array antenna shows measured reflection coefficients below −10 dB at 2.4 GHz and effective adjacent-element isolation. In addition, simulated results indicate that the GAF array antenna can perform beam scanning within the ±40° range, verifying the beam-control capability of this structure for UAV forward communication. Overall, this work highlights the feasibility of using GAF as a conductive material for both a high-efficiency radiator and an FSS beamforming structure, offering a practical material and design approach for lightweight, low-profile, and wing-conformal airborne array antennas.

## 1. Introduction

Unmanned aerial vehicles (UAVs) are increasingly used as airborne platforms for telemetry, data-link communication, navigation support, remote sensing, emergency response, environmental monitoring, and cooperative sensing [1,2,3]. These UAV-based airborne applications place coupled electromagnetic and platform-level requirements on airborne antenna systems, including reliable radiation and reception over the required operating bands, adequate angular coverage, low aerodynamic disturbance, reduced structural load, and compatibility with limited installation space. Conventional UAV and aircraft antenna systems often employ protruding monopoles, wire-frame antennas, blade antennas, or rigid externally mounted radiators because these structures can be connected to the radio-frequency (RF) feeding network and replaced with relative ease [4]. As the crucial interface for signal routing, the performance of this feeding network directly determines the impedance matching and power delivery efficiency of the airborne platform. However, as UAV airframes become smaller, lighter, and more integrated, such external antennas become less compatible with platform-level design. Their protruding profiles increase drag, their metallic supports add weight, and their installation can conflict with curved airframe surfaces and limited wing space.

These constraints have driven the transition from externally mounted airborne antennas to conformal antenna systems, in which radiating elements follow the shape of a cylindrical, conical, wing-like, or otherwise curved host surface instead of remaining on a planar or protruding support. When multiple such radiating elements are arranged on the curved surface, the resulting conformal array extends this concept to beam forming and scanning, and array theory provides the basis for preserving useful beam-forming capability on nonplanar platforms [5,6]. In radar and communication systems, conformal and phased-array antennas have long been investigated because they can provide wide angular coverage, a reduced profile, and closer integration with the platform body [7,8]. For UAV-oriented applications, recent studies have reported cylindrical and conical conformal arrays [9,10,11], flexible or wing-conformal arrays based on low-profile substrates and wing-integrated antenna skins [12,13,14,15,16], and pattern-diverse or compact onboard arrays for enhanced UAV communication [17,18]. Conformal array synthesis and radiation beam manipulation, namely the adjustment of the spatial gain distribution and radiation pattern through array geometry and element excitation, have also been examined for specialized airborne and hypersonic platforms [19,20]. Together, these studies indicate that conformal integration is increasingly used to reduce antenna profile while preserving angular coverage on airborne platforms.

Material selection remains important for lightweight conformal antennas because the conductive layer must provide electromagnetic performance while following curved airframe surfaces [13,16]. For UAV wing integration, the antenna material should combine high conductivity, low areal density, flexibility, environmental stability, and compatibility with thin polymer substrates. Copper conductors and rigid printed circuit boards are conventional options, but their weight and rigidity are not always favorable for low-profile conformal installation. A wing-mounted antenna may integrate radiators, directors, reflectors, and passive electromagnetic surfaces within a limited installation area. Therefore, using the same flexible conductor to support multiple electromagnetic functions is suitable for compact conformal integration.

Graphene-assembled film (GAF) offers a relevant material approach because graphene is widely recognized for its mechanical strength, flexibility, and electrical properties [21,22]. In practical microwave devices, highly conductive GAFs can approach metal-like electromagnetic behavior while retaining lower density, flexibility, and environmental stability [23]. GAF-based radiators and arrays have been explored for fifth-generation (5G) and millimeter-wave communication [24,25,26,27], electromagnetic shielding and multi-beam radiation [28], and flexible antennas ormetasurface-related functions [29,30,31]. These studies indicate that GAF can act not only as the conductive layer of an active radiator, but also as a patterned electromagnetic film for passive wave manipulation, extending its potential use from single radiating elements to integrated flexible antenna surfaces.

FSS structures and metasurfaces provide a compact approach for manipulating transmitted and reflected electromagnetic waves through engineered periodic unit cells. In this study, a periodic unit cell is defined as a single constituent pattern that is identically replicated and arranged periodically along the x- and y-directions in two dimensions to produce the required transmission or reflection response. In antenna systems, such surfaces have been used as transmissive layers, reflective layers, directors, and beam-shaping structures. Directive or transmissive FSS structures can narrow a feed beam and enhance forward directivity, whereas reflective FSS structures can redirect energy and modify the main-lobe direction without relying on a bulky metallic cavity or thick reflector. Related studies have shown the value of embedding conformal metasurfaces into structural platforms [32], reducing mutual coupling and controlling conformal-array beams [33,34,35], and achieving beam deflection, beam switching, or wide-coverage conformal operation in airborne and UAV-oriented arrays [36,37,38,39,40,41,42]. In this context, the trend toward integrated and adaptive wireless hardware further motivates lightweight multifunctional antenna structures [43].

In this work, we implement a lightweight and flexible S-band conformal antenna array based on highly conductive GAF for UAV wing platforms. The basic wing-conformal element consists of a GAF dipole radiator, a directive FSS, and a reflective FSS integrated on a polyimide (PI) substrate. This antenna is an actively fed radiating structure rather than a passive scattering surface under an externally incident wave. In this antenna configuration, the E-plane denotes the principal electric-field polarization plane of the dipole radiator, whereas the H-plane denotes the orthogonal magnetic-field plane. The directive and reflective FSS sections are excited by the field radiated from the dipole and are used to compress the E-plane beam and redirect the H-plane beam toward the forward sector. The element is further extended to a 1 × 4 GAF conformal array. In this design, four identical wing-conformal elements are aligned along the y-axis to form a linear array configuration with a uniform center-to-center element spacing of 0.5 $\lambda_{0}$, whose active impedance matching, port isolation, beam-scanning behavior, and radiation patterns are evaluated through full-wave simulation and prototype measurement. In this design, the same GAF/PI material platform is used for the active radiator and the passive beam-control structures, providing a low-profile route for wing-conformal antenna integration.

## 2. Materials and Experimental Methods

### 2.1. Graphene Film and Flexible Substrate

**Figure 1:**
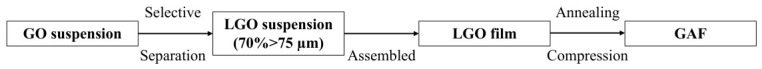
Schematic workflow for preparing the GAF used in this work.

A highly conductive GAF was used as the antenna conductor in this work. The film was prepared through high-temperature graphitization and mechanical densification, following GAF preparation routes and experimental characterization setups validated in previous highly conductive graphene-film microwave devices and wireless communication structures [27,29]. As shown in Figure 1, the fabrication of the highly conductive and mechanically robust GAF begins with synthesizing GO from a GO filter cake with a solid content of 43.1 wt%, supplied by Wuxi Chengyi (Wuxi, China), followed by preparing a GO suspension with a concentration of $15\;\mathrm{mg}\;{\mathrm{mL}}^{-1}$ and a seven-time fractionated centrifugation process of the GO suspension to isolate a large graphene oxide flake (LGO) precursor containing over 70% of flakes larger than 75 μm. This purified LGO suspension is uniformly blade-coated onto a polyethylene terephthalate (PET) substrate and dried at 70–80 °C, where the controlled flake concentration and drying temperature cooperate to form a uniform and continuous precursor film. In a high-temperature atmosphere furnace, the dried LGO film undergoes sequential carbonization at 1300 °C for 2 h and ultra-high-temperature graphitization at 2850 °C for 1 h. Finally, the graphitized film is subjected to mechanical rolling under a high pressure of 300 MPa, ultimately yielding a continuous, highly graphitized GAF with enhanced flexibility.

The flexible supporting layer was a PI film with a thickness of 0.05 mm, supplied by DuPont (Wilmington, DE, USA). The PI substrate had a relative permittivity of 3.5 and a loss tangent of 0.003. The loss tangent represents the dielectric loss factor of the PI substrate, and a lower value indicates smaller electromagnetic energy loss in the S-band antenna substrate. The use of an ultrathin PI film allowed the patterned GAF structures to conform to the wing profile without a rigid backing board or a thick spacer, consistent with prior PI-supported and wing-conformal antenna configurations for UAV-oriented platforms [13,14,16]. For the wing-conformal prototypes, the PI-supported GAF layer was attached to a foam wing-shaped support, which provided the mechanical curvature used in both the single-element and array-level measurements.

The prepared GAF was characterized before antenna fabrication using a combination of macroscopic and microscopic observations. The macroscopic appearance was recorded using a camera to document the overall film uniformity and metallic luster. The macroscopic flexibility was evaluated by manually bending the GAF to a large curvature and visually checking whether obvious wrinkles, cracks, or fracture appeared after bending. These photographs were used only for visual documentation of the sample’s appearance, bending state, and experimental configuration. Microscopic morphology and thickness information was obtained from field-emission scanning electron microscopy (FESEM, JEOL JSM-7610F Plus), as commonly used for highly conductive graphene-film characterization [23,28]. Before SEM observation, a small piece of GAF was cut from the prepared film, and its exposed cross-section was fixed on the SEM sample stage for imaging. The FESEM images were acquired at an accelerating voltage of 5 kV under low-vacuum mode to reduce charging effects. The SEM observation focused on the layered cross-sectional structure, local wrinkles, cracks, and interlayer stacking. This characterization was used to relate the conductive film preparation process to the subsequent laser-patterned dipole and FSS structures.

### 2.2. Antenna Fabrication

**Figure 2:**
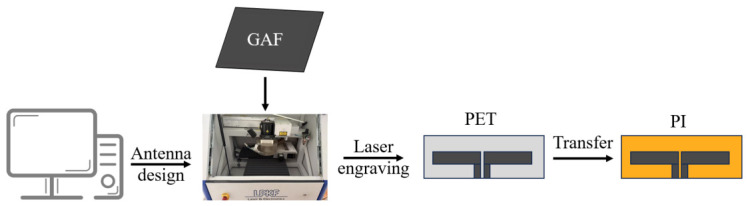
GAF antenna fabrication process.

The laser patterning fabrication of the GAF layout and the SubMiniature version A (SMA) coaxial feeding technique used in this study are similar to the graphene antenna fabrication processes [27,29]. An LPKF ProtoLaser S system equipped with a diode-pumped laser was used for the patterning process. The laser system uses a 1064 nm near-infrared source and provides an adjustable pulse repetition frequency of 10–100 kHz. Its focused spot diameter is no larger than 25 μm, and the laser scanning accuracy and repeatability are both no larger than 2 μm. The working area is at least 200 mm × 300 mm, and the area processing rate is no less than 6 cm^2^/min for rapid prototyping. The laser-patterning precision was 10 μm. As shown in Figure 2, the fabrication of the GAF-based antenna begins with transferring the optimized digital antenna layout from a computer to a precision laser engraving machine, which selectively cuts and etches the GAF to pattern the exact antenna geometries. The precisely engraved GAF antenna pattern is initially formed on a temporary polyethylene terephthalate (PET) carrier substrate to preserve its fine structural gaps and alignment, and is subsequently transferred onto a flexible PI target dielectric film, ultimately yielding the low-profile and mechanically robust GAF/PI flexible antenna.

For the single wing-conformal antenna element, the patterned GAF/PI sheet was bonded onto the wing-shaped foam support, consistent with prior conformal antenna prototypes mounted on curved or wing-like supports [13,14,16]. The dipole was fed through a SMA connector and a 50 Ω coaxial line. The inner conductor of the coaxial line was connected to one dipole arm, while the outer conductor was connected to the other arm. The same feeding method was used for each element in the 1 × 4 conformal array. In the array prototype, four identical GAF wing-conformal elements were arranged along the spanwise direction, and each port was equipped with an individual SMA feed so that reflection coefficients, transmission coefficients, and element-by-element radiation patterns could be measured.

### 2.3. Simulation and Measurement Methods

Full-wave simulations were performed using CST Studio Suite 2023, a commercial full-wave electromagnetic simulation software package from Dassault Systèmes. The modeling procedure was performed in four steps. First, the GAF conductor, PI substrate, foam support, dipole radiator, and square-ring FSS unit cells were defined using the measured material parameters and optimized geometric dimensions. Second, parameter sweeps were used to tune the directive and reflective FSS unit cells. Third, the optimized dipole and FSS layers were assembled on the wing-shaped support to form the single wing-conformal element. Finally, four identical elements were arranged along the spanwise direction to construct the 1 × 4 conformal array, with each element assigned an individual 50 Ω port. Time-domain full-wave simulation was used for the radiator, conformal element, and array. Open boundary conditions were adopted for radiation analysis. For the array model, the simulation band was set to 2.0–2.8 GHz, and local mesh refinement was applied near feed gaps and GAF edges to improve the resolution of geometrically sensitive regions.

The antenna optimization was carried out sequentially. The dipole dimensions, including the arm length, arm width, and feed gap, were first adjusted to obtain impedance matching around 2.4 GHz. The directive and reflective FSS unit-cell dimensions and their placement relative to the dipole were then swept to enhance forward radiation, compress the E-plane beam, and redirect the H-plane beam toward the wing-front sector. For the 1 × 4 array, the element spacing and excitation amplitudes were selected to preserve port matching while narrowing the main beam and controlling the sidelobe level.

**Figure 3:**
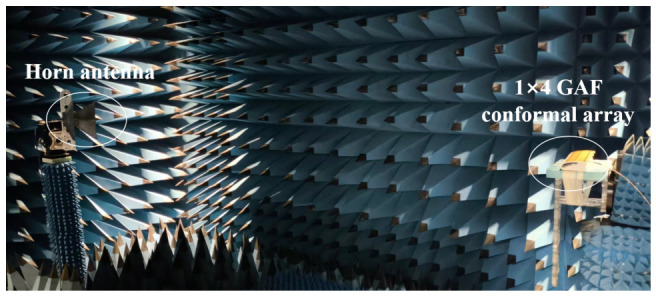
Microwave anechoic chamber setup used for measuring the 1 × 4 GAF conformal array.

The proposed dipole geometry and its impedance response are presented in Figure 5, where the schematic shows the optimized arm length, strip width, and feed region used for the baseline radiator. The simulated reflection coefficient was obtained from the 50 Ω coaxial port in CST Studio Suite after the dipole dimensions were optimized. The reflection coefficients of the fabricated dipole and wing-conformal element were then measured using a vector network analyzer (VNA). A Keysight PNA N5247A VNA was used for these measurements. Before measurement, the VNA was calibrated at the end of the coaxial test cable, and the measured $S_{11}$ was recorded as the reflection coefficient of the connected antenna port. Four conformal GAF antenna elements are arranged in a straight line to form a 1 × 4 GAF conformal array, and each element is equipped with an SMA connector for antenna feeding. All-port reflection coefficients and inter-port transmission coefficients were measured using a Keysight PNA N5247A VNA to evaluate port matching and element isolation under a finite phased-array environment. Far-field radiation measurements of this 1 × 4 configuration were conducted in a microwave anechoic chamber with physical dimensions of 5.6 m × 5.0 m × 3.8 m, as schematically illustrated in Figure 3. Within this testing environment, the fabricated 1 × 4 GAF conformal array served as the transmitting antenna on a computer-controlled rotation platform, while a 1–20 GHz double-ridged horn antenna was utilized as the fixed receiver. Measurement data was obtained by precisely measuring the reception of the RF signal at the receiving antenna using a VNA, thus determining the far-field radiation performance of the GAF conformal array antenna. To examine the pattern differences dictated by the finite-array coupling environment, H-plane active element patterns were recorded sequentially at a representative S-band frequency of 2.4 GHz. This frequency was selected as a representative S-band operating point because it lies within the impedance-matched band of the fabricated antenna and is relevant to common UAV telemetry and data-link applications. Therefore, measuring the H-plane normalized radiation patterns at 2.4 GHz provides a consistent basis for comparing the forward radiation behavior of the four array elements. During each individual element measurement, the specific target port was excited via its SMA line while the remaining three adjacent ports were capped with 50 Ω matched loads to accurately emulate the inactive termination states of a multi-port antenna skin.

## 3. Results and Discussion

### 3.1. GAF Film Characterization

**Figure 4:**
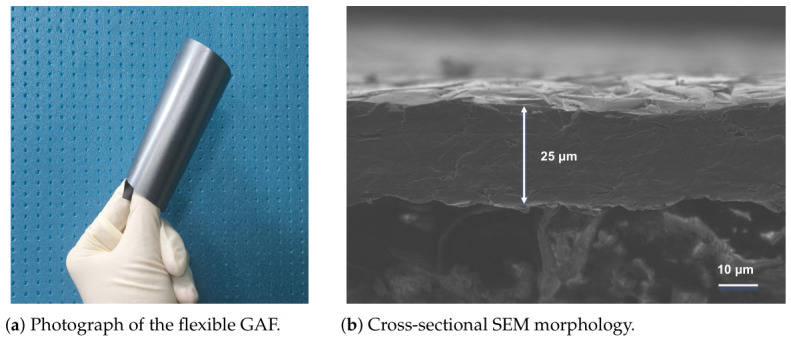
Material characterization of the GAF conductor: (**a**) digital photograph of the flexible graphene film and (**b**) cross-sectional SEM image showing the layered morphology and a film thickness of approximately 25 μm.

The digital photograph of the GAF shown in Figure 4a presents a film with a metallic luster, and the rolled state indicates good macroscopic flexibility. The cross-sectional SEM image in Figure 4b shows a compact layered morphology with an overall thickness of approximately 25 μm. The cross-section is continuous across the observed region, and no obvious large voids, cracks, or delamination are observed, indicating acceptable morphological homogeneity for subsequent laser patterning and conformal attachment. Local wrinkles and stacked graphene layers can be observed in the film cross-section, which are associated with the high-temperature thermal reduction, graphitization, and subsequent densification process. These local features reflect the intrinsic laminated structure of densified graphene films rather than macroscopic structural nonuniformity. This wrinkled and layered structure can accommodate bending deformation and is beneficial for maintaining the structural integrity of the GAF during flexible conformal integration.

### 3.2. GAF Wing-Conformal Antenna Element

#### 3.2.1. Dipole Radiator Baseline

**Figure 5:**
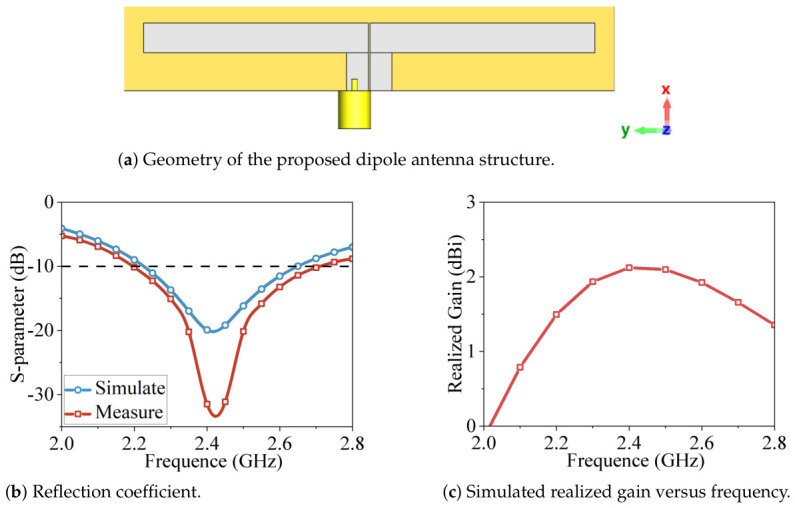
Geometry and impedance response of the GAF dipole radiator: (**a**) Geometry of the proposed dipole antenna structure, (**b**) Reflection coefficient, and (**c**) simulated realized gain.

Dipole antennas are widely used as basic radiating elements because of their simple geometry, well-understood current distribution, and broad radiation behavior. Their planar structure also facilitates integration with director- or reflector-type passive surfaces. Therefore, a GAF dipole antenna was adopted as the active radiating element in this work. As shown in Figure 5a, the dipole adopts a coaxially fed half-wave configuration. After parameter optimization, the main dimensions were set as L=59.8 mm, w=3.9 mm, and $w_{1}=2.9$ mm. The feed gap and arm width were adjusted to obtain impedance matching while retaining a simple planar geometry suitable for subsequent conformal integration.

The simulated reflection coefficient in Figure 5b reaches −19.9 dB at 2.4 GHz, and the simulated −10 dB impedance bandwidth extends from 2.23 to 2.64 GHz. The fabricated dipole shows a measured resonance near the same frequency, with a measured reflection coefficient of about −31.4 dB at 2.4 GHz and a −10 dB impedance bandwidth of 2.2–2.7 GHz. The measured response is slightly broader than the simulated one, which can be attributed to fabrication tolerance, feed soldering, and the practical coaxial transition. Nevertheless, the simulated and measured responses follow the same trend and support the use of the patterned GAF dipole as the active radiator in the S-band design.

**Figure 6:**
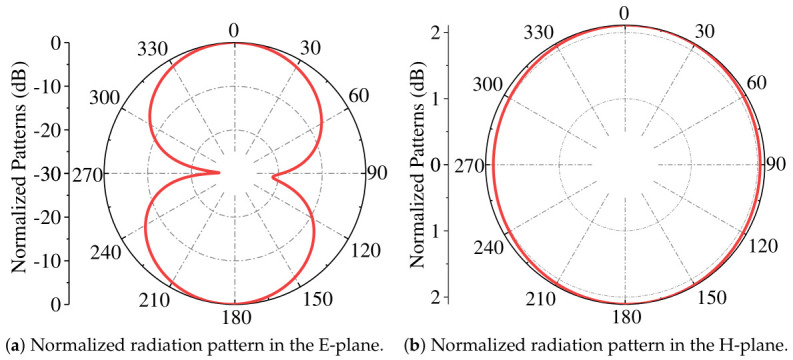
Normalized radiation patterns of the GAF dipole radiator: (**a**) E-plane and (**b**) H-plane.

The simulated realized gain of the dipole is 2.12 dBi at 2.4 GHz, as shown in Figure 5c. The E-plane pattern in Figure 6a exhibits the expected dipole-like bidirectional response, whereas the H-plane pattern in Figure 6b remains close to omnidirectional. The reported E-plane and H-plane radiation patterns correspond to the designed operating polarization of the actively fed dipole radiator, and no external incident polarization is introduced in this antenna radiation evaluation. This wide-beam response provides the baseline radiation behavior and indicates that additional beam shaping is needed when a more concentrated wing-front radiation direction is required.

#### 3.2.2. Directive FSS Design and Simulation

**Figure 7:**
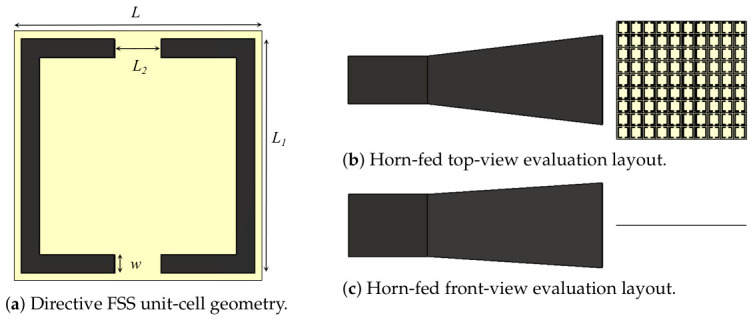
Directive FSS geometry and horn-fed evaluation model: (**a**) unit-cell geometry, (**b**) top-view layout, and (**c**) front-view layout.

A directive FSS was then introduced to reduce the 3 dB beamwidth of the main lobe and enhance forward radiation. The unit cell is a split square-ring GAF pattern, as shown in Figure 7a. The optimized parameters were L=23 mm, $L_{1}=22$ mm, $L_{2}=5$ mm, and w=2 mm. Similarly to the director elements used in Yagi-type antennas, the directive FSS is arranged in front of the feed so that the radiated field is reinforced toward the FSS side. The simple ring-type geometry also allows the unit cell to be laser patterned using the same GAF conductor as the dipole.

**Figure 8:**
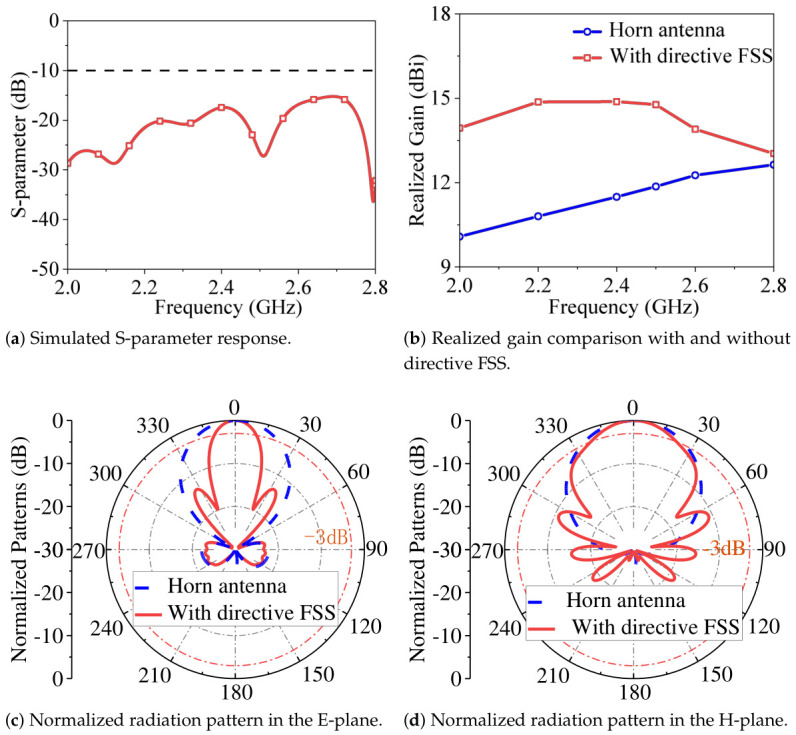
Simulated response and beam-compression performance of the directive FSS: (**a**) S-parameter response, (**b**) realized gain comparison, and (**c**,**d**) normalized radiation patterns.

For independent evaluation of its beam-compression function, the unit cell was expanded into a 10 × 9 FSS array and placed 24 mm in front of a standard-gain horn, as shown in Figure 7b,c. This horn-fed model separates the FSS response from the later effects of dipole feeding, wing curvature, and finite wing installation. Figure 8a shows the simulated S-parameter response of the directive FSS evaluation model. In the gain and pattern plots, “Horn antenna” denotes the reference feed without FSS loading, whereas “With directive FSS” denotes the same feed after the directive FSS is introduced.

After loading the directive FSS, the realized gain increases from 11.49 to 14.88 dBi at 2.4 GHz, as shown in Figure 8b. The E-plane pattern in Figure 8c shows that the 3 dB beamwidth of the main lobe is reduced from 43.9° to 20.9°. The H-plane beamwidth in Figure 8d is also reduced from 54.1° to 43.2°, although the narrowing effect is less pronounced than that in the E-plane. In this article, the H-plane 3 dB beamwidth refers to the angular width of the main beam on the H-plane radiation pattern when it is 3 dB below its peak value. Its reduction means that the radiation distribution is more concentrated in the expected forward direction and the radiation capability is stronger. These results indicate that the directive FSS mainly functions as a transmission-type beam-narrowing surface and provides the forward directivity needed for the subsequent conformal element.

#### 3.2.3. Reflective FSS Design and Simulation

**Figure 9:**
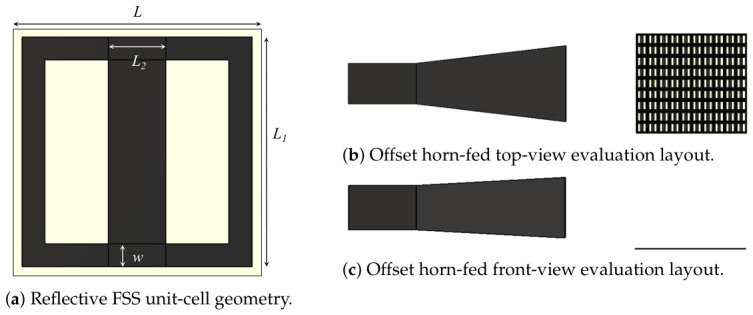
Reflective FSS geometry and offset horn-fed evaluation model: (**a**) unit-cell geometry, (**b**) top-view layout, and (**c**) front-view layout.

A reflective FSS was further designed to modify the H-plane radiation direction. As shown in Figure 9a, the unit cell is based on a square-ring GAF structure with loaded strips inside the ring. The additional strips increase the electrical length of the cell and help tune the reflective response near the operating frequency. The optimized geometric parameters were L=22 mm, $L_{1}=20$ mm, $L_{2}=5$ mm, and w=2 mm. In the wing-conformal configuration, this surface is used as a reflector-like FSS layer to redirect part of the radiated energy toward the wing-front direction.

**Figure 10:**
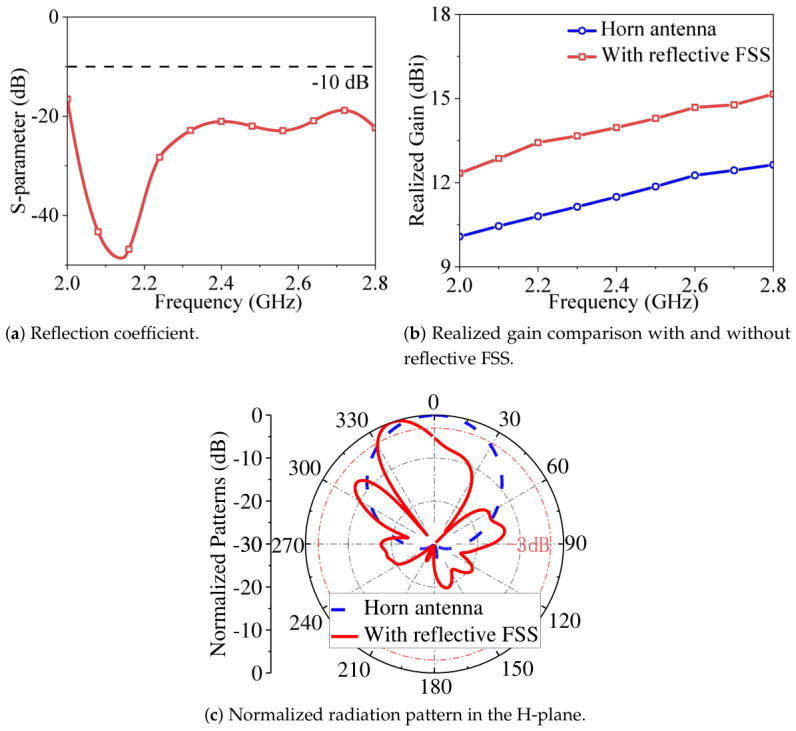
Simulated response and beam-redirection performance of the reflective FSS: (**a**) Reflection coefficient, (**b**) realized gain comparison, and (**c**) normalized radiation pattern.

The reflective FSS was also evaluated using a horn-fed model. A 10 × 9 reflective FSS array was placed 140 mm from the horn aperture and vertically offset by 27 mm, as shown in Figure 9b,c. The offset arrangement was adopted to examine whether the FSS could redirect the main lobe rather than merely increase broadside gain. Figure 10a shows the simulated reflective response of the reflective FSS evaluation model, and Figure 10b,c compare the reference horn with the reflective-FSS-loaded horn.

With the reflective FSS, the realized gain increases from 11.49 to 13.94 dBi at 2.4 GHz. In addition, the H-plane main lobe is deflected and narrowed. The 3 dB beamwidth of the main lobe is reduced from 54.1° for the reference horn to 22.2° after the reflective FSS is introduced. This behavior is consistent with the expected function of a reflector-like FSS surface: it modifies the reflected field distribution and tilts the main beam toward the desired angular region.

#### 3.2.4. Integrated Wing-Conformal Antenna Element

**Figure 11:**
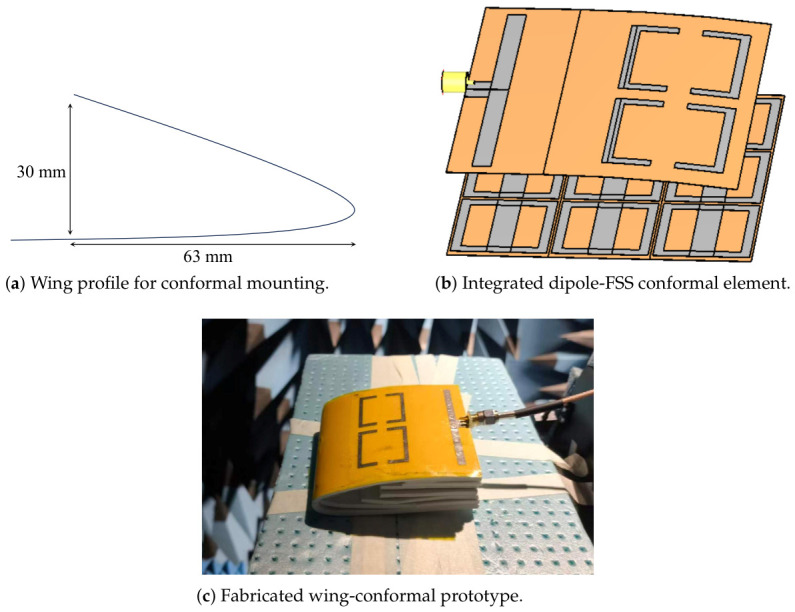
Geometry and prototype of the GAF wing-conformal antenna element: (**a**) wing profile for conformal mounting, (**b**) integrated dipole-FSS conformal element, and (**c**) fabricated prototype.

After the dipole, directive FSS and reflective FSS were characterized separately, and the three parts were integrated into a single wing-conformal element. Figure 11a shows the wing profile used as the conformal support, and Figure 11b shows the integrated layout. The directive FSS was placed in front of the dipole along the intended radiation direction, while the reflective FSS was arranged on the lower wing region to support H-plane beam redirection. To fit the available wing surface, the directive section was reduced to two linearly arranged units, each approximately 27 mm × 27 mm, and the reflective section was arranged as three linearly distributed units, each approximately 65 mm × 65 mm. The fabricated prototype is shown in Figure 11c.

**Figure 12:**
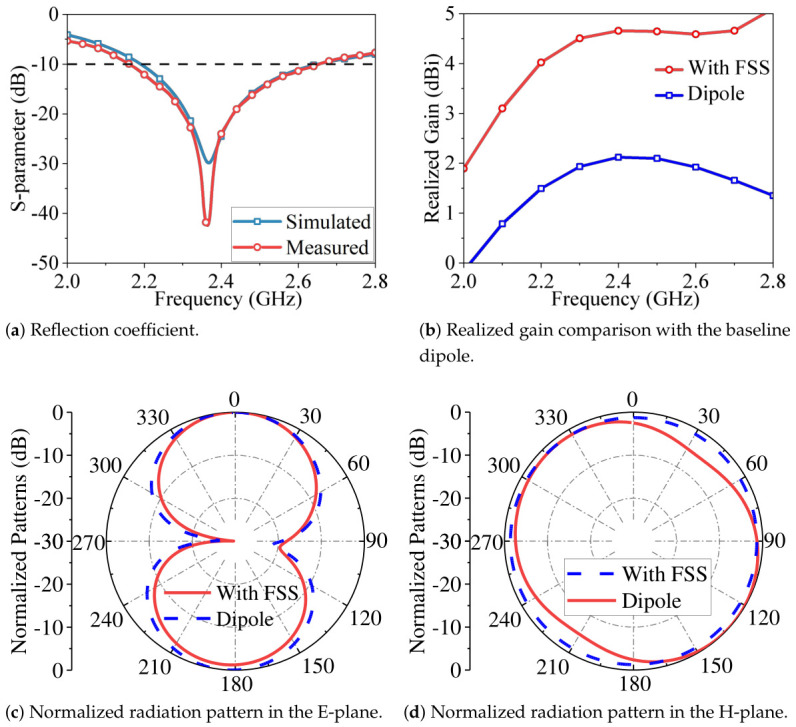
Measured and simulated performance of the GAF wing-conformal antenna element: (**a**) Reflection coefficient, (**b**) realized gain comparison with the baseline dipole, and (**c**,**d**) normalized radiation patterns.

The reflection coefficients in Figure 12a show that the resonance is preserved after the GAF/PI sheet is mounted on the wing-shaped support. The simulated reflection coefficient is −21.3 dB at 2.4 GHz, and the simulated −10 dB bandwidth is 2.19–2.65 GHz. The fabricated conformal element shows a measured reflection coefficient of about −24 dB at 2.4 GHz, with a measured −10 dB bandwidth of 2.16–2.65 GHz. The agreement between simulation and measurement indicates that the flexible GAF/PI conductor and foam-supported wing curvature do not introduce a severe impedance shift.

The radiation results in Figure 12b–d compare the bare GAF dipole with the integrated wing-conformal element. After FSS loading, the realized gain increases from 2.12 to 4.65 dBi at 2.4 GHz. The E-plane pattern maintains a dipole-like form, which indicates that the active radiator remains the dominant source of radiation. In contrast, the H-plane pattern changes from a nearly omnidirectional response to an elliptical and tilted main-lobe distribution. This result shows that the compact directive and reflective FSS sections retain their beam-shaping roles after being reduced from the larger horn-fed evaluation arrays to a wing-compatible conformal layout.

### 3.3. 1 × 4 GAF Conformal Array

#### 3.3.1. Array Geometry and Simulated Performance

**Figure 13:**
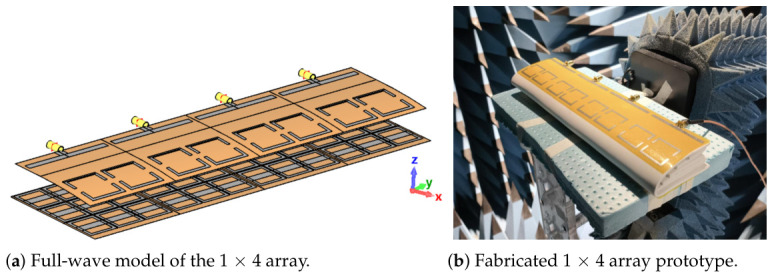
Geometry and prototype of the 1 × 4 GAF conformal array: (**a**) full-wave model and (**b**) fabricated prototype.

**Figure 14:**
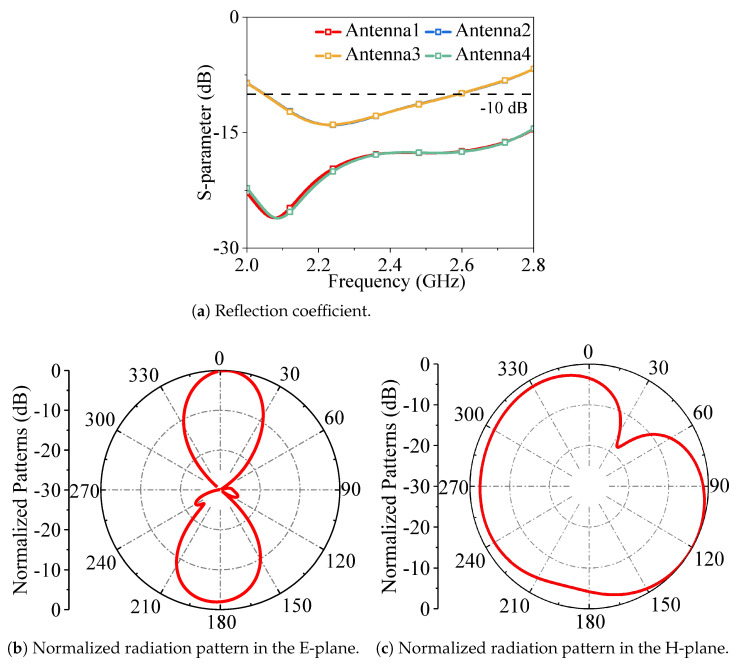
Simulated performance of the 1 × 4 GAF conformal array: (**a**) Reflection coefficient and (**b**,**c**) normalized radiation patterns.

A 1 × 4 conformal array was constructed by arranging four identical wing-conformal elements along the spanwise direction. Each element retained the GAF dipole, directive FSS, and reflective FSS configuration described above. The overall array dimensions were 260 mm × 63 mm × 30.075 mm. To obtain a narrower main lobe while controlling the sidelobe level, the array adopted in-phase non-uniform excitation based on Taylor synthesis, with a port amplitude ratio of 1:0.363. Figure 13 shows the simulated array geometry and fabricated prototype, while Figure 14 gives the simulated array-level responses.

The 1 × 4 array was analyzed using a multi-port full-wave model over 2.0–2.8 GHz. In this model, all four radiating elements and their ports were included simultaneously, so the simulated impedance response accounts for the mutual coupling between adjacent and non-adjacent elements. The four reflection coefficient traces in Figure 14a correspond to Antenna 1–Antenna 4. All four elements maintain comparable impedance behavior near the operating frequency, indicating that the single-element matching is not strongly degraded by the finite array environment. The active voltage standing wave ratios (active VSWRs) extracted at 2.4 GHz were 1.23, 1.28, 1.27, and 1.18 for Ports 1–4, respectively. Across 2.27–2.59 GHz, the active VSWRs remain below 1.8, showing that the array preserves useful active impedance matching around the target band.

The simulated E-plane and H-plane patterns are shown in Figure 14b,c. In the E-plane, the 3 dB beamwidth of the main lobe is 32°, and the sidelobe level is about −12 dB under the adopted amplitude taper. In the H-plane, the main lobe points approximately toward ϕ ≈ 22°, which is consistent with the beam-tilting behavior introduced by the reflective FSS in each element.

**Figure 15:**
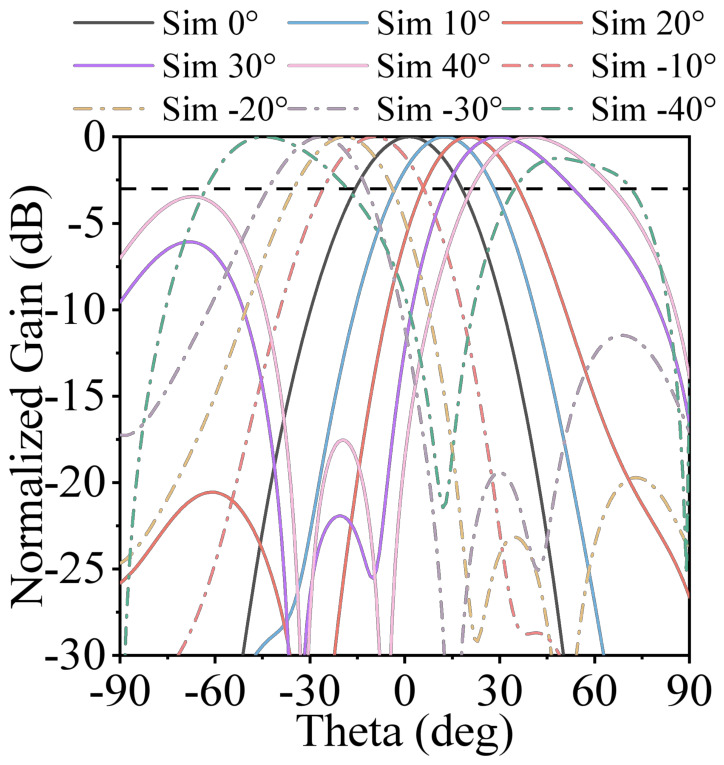
Simulated normalized radiation patterns of the 1 × 4 GAF conformal array under progressive phase excitation from −40° to 40°. The dashed horizontal line indicates the −3 dB reference level.

Progressive phase excitations were further applied in the full-wave array model to evaluate beam-scanning capability. The normalized scanning patterns in Figure 15 cover scan angles from −40° to 40° with a 10° interval. The main lobe is defined as the dominant radiation beam corresponding to the maximum gain direction in each normalized pattern. This dominant beam follows the prescribed scan direction across the considered angular range. At the outer scan states, the lobes become broader and the patterns show stronger distortion because of the finite aperture and conformal element environment. Since the curves are normalized, this figure emphasizes beam-steering behavior and relative beam shape rather than absolute scan loss. These results support the capability of the GAF conformal array to provide directional radiation and controlled angular coverage.

#### 3.3.2. Array Prototype and Measured S-Parameters

**Figure 16:**
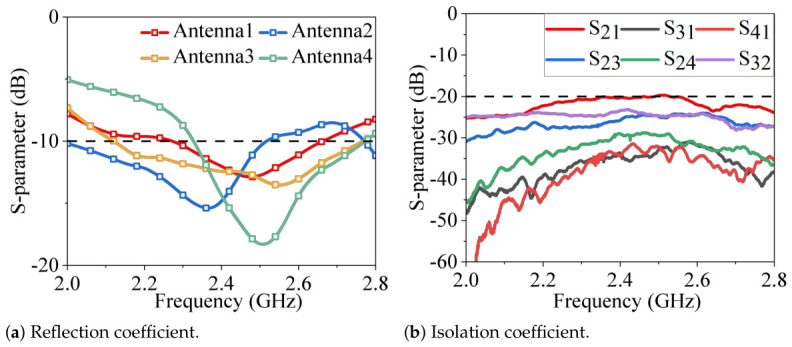
Measured multi-port S-parameters of the fabricated 1 × 4 GAF conformal array: (**a**) Reflection coefficient and (**b**) Isolation coefficient.

The 1 × 4 GAF conformal array prototype was fabricated using the same laser-patterned GAF/PI process as the single element. As shown in Figure 13b, the flexible array sheet was attached to the foam wing support, and each element was fed by an individual SMA connector and 50 Ω coaxial line. The fabricated finite array prototype was then measured using a VNA to characterize both port matching and inter-element coupling. Figure 16 gives the measured multi-port S-parameters.

The measured reflection coefficients in Figure 16a are below the −10 dB matching criterion at 2.4 GHz for all four ports. The measured values are −12.15, −14.97, −11.75, and −14.75 dB for Ports 1–4, respectively. The small differences among the four ports are mainly associated with the finite array boundary, fabrication tolerance, and SMA assembly. The measured transmission coefficients in Figure 16b quantify inter-port coupling through the two-port S-parameters $S_{ij}$ measured by the VNA, with the unused ports terminated by matched loads. Lower $|S_{ij}|$ values indicate weaker coupling and higher isolation between the corresponding ports. Adjacent-port isolation is about −20 to −24 dB, while non-adjacent isolation improves to about −25 to below −34 dB. The isolation trend follows the expected reduction in coupling with increasing element separation and supports the feasibility of the fabricated multi-port conformal array.

#### 3.3.3. Measured H-Plane Patterns

**Figure 17:**
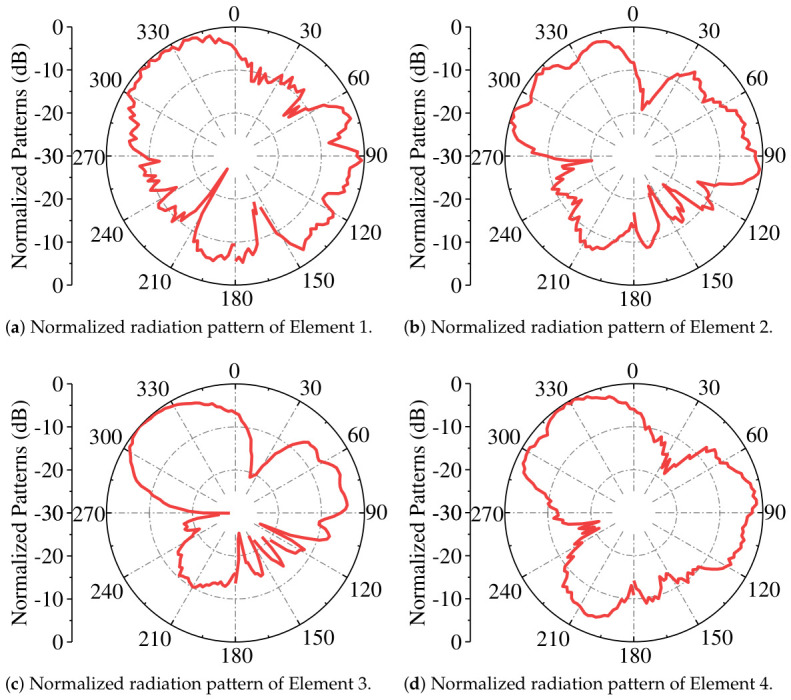
Measured H-plane normalized radiation patterns of the four elements in the fabricated 1 × 4 GAF conformal array: (**a**) Element 1, (**b**) Element 2, (**c**) Element 3, and (**d**) Element 4. Each element was measured under individual excitation, while the remaining ports were terminated with matched loads.

The radiation behavior of the fabricated finite array was evaluated through element pattern measurements. During each measurement, one element was excited and the remaining ports were terminated with matched loads. This procedure is consistent with the active-element-pattern method commonly used for finite phased arrays, and it reveals how each element radiates in the presence of neighboring elements and the conformal support. Figure 17 shows the measured H-plane patterns of the four array elements.

All four measured patterns retain a main beam toward the wing-front direction. This result indicates that the beam-redirection effect observed for the single element is preserved after the elements are integrated into the fabricated array. The edge elements show relatively smooth patterns because each edge element is mainly affected by one neighboring element. By contrast, the center elements exhibit stronger pattern distortion because they are coupled to neighboring elements on both sides. This trend is consistent with the coupling environment of a finite linear array. Although simultaneous-excitation array patterns were not measured in the current prototype, the measured patterns and S-parameters together indicate that the GAF/PI conformal array retains forward radiation behavior while maintaining port matching and inter-element isolation at the element level.

### 3.4. Material Integration and Technical Implications

The same highly conductive GAF conductor is used throughout the active dipole, directive FSS, reflective FSS, and finite conformal array. This material continuity is useful for wing-conformal integration because the radiating conductor and the passive beam-control patterns can be fabricated on one flexible film and then supported by the same ultrathin PI substrate. As a result, the antenna element avoids a stack of separate metallic radiators, directors, and reflectors while retaining the low-profile attachment needed for the wing-shaped support.

The electromagnetic function is built in stages. The GAF dipole establishes the S-band resonance and baseline radiation. The directive FSS narrows the main-lobe beamwidth and enhances gain, while the reflective FSS provides H-plane beam tilting. After these two FSS sections are incorporated into the conformal element, the measured operating band of 2.16–2.65 GHz and the simulated gain of 4.65 dBi show that the compact wing-compatible layout retains the main functions observed in the separate FSS evaluation models. The 1 × 4 array further extends this material and structural scheme from a single conformal element to a multi-port conformal array, with measured adjacent isolation below −20 dB and simulated ±40° beam-scanning capability.

This geometry offers a low-profile and wing-compatible array layout while allowing the same flexible GAF platform to support both radiation and passive beam control. Its main limitation is that the finite 1 × 4 aperture and curved mounting surface can broaden or distort the pattern at larger scan angles.

Recent graphene-based metasurface studies have shown that tunable polarization conversion surfaces can be used to reduce the radar cross-section of array antennas by controlling the polarization state and scattering behavior of reflected waves [44]. This development illustrates the broader role of graphene-based electromagnetic surfaces in multifunctional antenna systems. Differing from such tunable scattering-control metasurfaces, the present work uses a passive, highly conductive GAF platform to integrate an active dipole radiator with directive and reflective FSS beam-control sections on a flexible wing-conformal substrate. The emphasis is therefore placed on material continuity, low-profile conformal integration, and forward radiation control for UAV-oriented S-band antenna arrays.

## 4. Conclusions

This work demonstrates a lightweight S-band wing-conformal array antenna based on a highly conductive graphene-assembled film. The first contribution is the use of flexible, highly conductive GAF as the antenna conductor together with a flexible PI dielectric substrate to form a GAF-based conformal wing antenna. This material and substrate combination supports an antenna configuration with a low profile, low weight, and good conformability on curved UAV wing surfaces.

The second contribution is the design of a GAF conformal antenna element in which a dipole radiator is combined with directive and reflective FSS structures. The directive FSS provides forward beam compression, while the reflective FSS redirects the H-plane radiation toward the intended wing-front sector, enabling effective beam control and stable directional radiation at 2.4 GHz. The element is further extended to a 1 × 4 multi-port conformal array. Prototype measurements and full-wave simulations verify that the array maintains port matching, element isolation, forward radiation behavior, and beam-steering capability in the target S-band. These results indicate that highly conductive GAF can serve as a multifunctional flexible conductor for conformal antenna arrays, offering a feasible route toward lightweight, integrated, and beam-controllable antenna skins for UAV and other unmanned platforms. Future work may further combine flexible conformal arrays with data-driven modeling methods, such as machine learning-assisted prediction of nonlinear graphene responses [45], to support material-related parameter optimization, field-dependent performance analysis, and adaptive electromagnetic design.

## Data Availability

The original contributions presented in this study are included in the article. Further inquiries can be directed to the corresponding authors.
